# Bis(2-meth­oxy-6-{[2-(methyl­ammonio)eth­yl]imino­meth­yl}phenolato)thio­cyanato­zinc(II) thio­cyanate hemihydrate

**DOI:** 10.1107/S1600536809022326

**Published:** 2009-06-20

**Authors:** Sanjun Peng, Fen Zhang

**Affiliations:** a Department of Chemistry and Biological Engineering, Changsha University of Science and Technology, Changsha 410014, People’s Republic of China; b School of Foreign Language, Jiangsu University, Jiangsu 212013, People’s Republic of China

## Abstract

The title mononuclear zinc(II) complex, [Zn(C_11_H_16_N_2_O_2_)_2_(NCS)]NCS·0.5H_2_O, consists of a complex cation, a thio­cyanate anion, and half of a water mol­ecule. The Zn^II^ atom in the cation is five-coordinated by two imine N and two phenolate O atoms from two bidentate Schiff base ligands, and by one N atom of a thio­cyanate ligand, forming a distorted trigonal-bipyramidal geometry. The ammonio H atoms are involved in hydrogen bonding with the ligand O atoms and the solvent water molecules (site occupation factor 0.5), which partially determines the conformation of the ligands.

## Related literature

For background to the properties of zinc(II) complexes, see: Lipscomb & Sträter (1996 ▶); Bertini *et al.* (1994 ▶); Harrison *et al.* (2006 ▶); Tirosh *et al.* (2005 ▶); Musie *et al.* (2004 ▶); Vallee & Auld (1993 ▶). For related structures, see: Li *et al.* (2008 ▶); Eltayeb *et al.* (2008 ▶); Zhang & Wang (2007 ▶); Cai (2009 ▶).
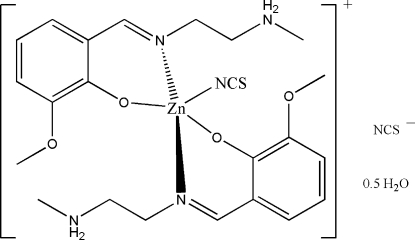


## Experimental

### 

#### Crystal data

[Zn(C_11_H_16_N_2_O_2_)_2_(NCS)]NCS·0.5H_2_O
*M* *_r_* = 607.06
Triclinic, 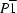

*a* = 9.997 (2) Å
*b* = 13.017 (3) Å
*c* = 13.379 (3) Å
α = 73.70 (3)°
β = 77.95 (3)°
γ = 72.25 (3)°
*V* = 1577.0 (5) Å^3^
*Z* = 2
Mo *K*α radiation
μ = 0.95 mm^−1^
*T* = 298 K
0.30 × 0.28 × 0.27 mm

#### Data collection

Bruker SMART APEX CCD area-detector diffractometer
Absorption correction: multi-scan (*SADABS*; Bruker, 2000 ▶) *T*_min_ = 0.764, *T*_max_ = 0.784
12467 measured reflections
6300 independent reflections
3199 reflections with *I* > 2σ(*I*)
*R*_int_ = 0.057

#### Refinement

*R*[*F* ^2^ > 2σ(*F* ^2^)] = 0.081
*wR*(*F* ^2^) = 0.190
*S* = 0.99
6300 reflections
346 parameters
18 restraints
H-atom parameters constrained
Δρ_max_ = 0.51 e Å^−3^
Δρ_min_ = −0.37 e Å^−3^

### 

Data collection: *SMART* (Bruker, 2000 ▶); cell refinement: *SAINT* (Bruker, 2000 ▶); data reduction: *SAINT*; program(s) used to solve structure: *SHELXTL* (Sheldrick, 2008 ▶); program(s) used to refine structure: *SHELXTL*; molecular graphics: *SHELXTL*; software used to prepare material for publication: *SHELXTL*.

## Supplementary Material

Crystal structure: contains datablocks global, I. DOI: 10.1107/S1600536809022326/su2115sup1.cif

Structure factors: contains datablocks I. DOI: 10.1107/S1600536809022326/su2115Isup2.hkl

Additional supplementary materials:  crystallographic information; 3D view; checkCIF report

## Figures and Tables

**Table 1:** Hydrogen-bond geometry (Å, °)

*D*—H⋯*A*	*D*—H	H⋯*A*	*D*⋯*A*	*D*—H⋯*A*
N4—H4*B*⋯O2	0.90	2.38	3.001 (8)	126
N4—H4*B*⋯O1	0.90	1.84	2.682 (7)	155
N4—H4*A*⋯O5	0.90	1.99	2.839 (14)	157
N2—H2*B*⋯N6	0.90	1.93	2.834 (11)	179
N2—H2*A*⋯O4	0.90	2.35	2.889 (8)	119
N2—H2*A*⋯O3	0.90	1.86	2.691 (7)	153

**Fig. 1.**
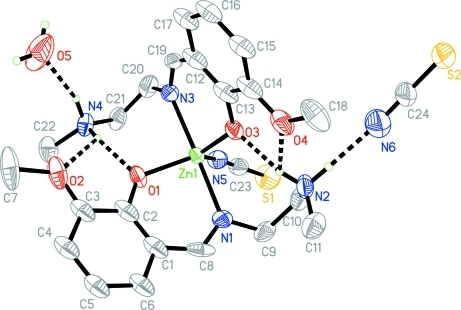
The molecular structure of the title compound, with displacement ellipsoids drawn at the 30% probability level.

## supplementary crystallographic information

### Comment

Zinc is the second most abundant transition metal in biology, functions as the active site of hydrolytic enzymes, such as carboxypeptidase and carbonic anhydrase, where it is in a hard donor coordination of nitrogen and oxygen (Lipscomb & Sträter, 1996; Bertini *et al.*, 1994). Zinc atom can readily adopt four-, five- or six-coordination (Harrison *et al.*, 2006; Tirosh *et al.*, 2005; Musie *et al.*, 2004; Vallee & Auld, 1993). As a continuation of work on this area, we report herein the new title zinc(II) complex, with the Schiff base 2-methoxy-6-[(2-methylaminoethylimino)methyl]phenol.

The title compound consists of a complex cation, a thiocyanate anion, and a half water molecule of crystallization (Fig. 1). The Zn^II^ atom in the cation is five-coordinated by two imine N and two phenolate O atoms, from two Schiff base ligands, and by one N atom of a thiocyanate ligand, so forming a trigonal-bipyramidal geometry. The amine N atom is protonated and does not coordinate to the metal ion. The NH_2_^+^ hydrogen atoms are involved in hydrogen bonding with the ligand O-atoms which partially determines the conformation of the ligands. The Zn—O and Zn—N bond lengths [1.977 (4) - 1.979 (4) Å and 2.001 (6) - 2.135 (5) Å, respectively] are comparable to the values in similar complexes (Li *et al.*, 2008; Eltayeb *et al.*, 2008; Zhang & Wang, 2007; Cai, 2009).

### Experimental

3-Methoxysalicylaldehyde (0.1 mmol, 15.2 mg) and *N*-methylethane-1,2-diamine (0.1 mmol, 7.4 mg) were stirred into 30 ml of methanol. After 1 h, ammonium thiocyanate (0.1 mmol, 7.6 mg) in water (1 ml) and zinc acetate (0.1 mmol, 22.0 mg) in methanol (10 ml) was added, and the stirring continued for a further 1 h. The filtrate was kept at rt for about a week, depositing colorless block-like crystals of the title compound.

### Refinement

All  H atoms were positioned geometrically and refined as riding atoms: C—H = 0.93–0.97 Å, N—H = 0.90 Å, O—H = 0.85 Å, with *U*_iso_(H) set to 1.2*U*_eq_(C/O) and 1.5*U*_eq_(methyl C). The structure contains solvent accessible voids of 138.00 A^3^, which might accommodate a disordered water molecule. The phenyl rings were refined as regular hexagons, with their ADP's made equal to one another.

### Crystal data

**Table:**

[Zn(C_11_H_16_N_2_O_2_)_2_(NCS)]NCS·0.5H_2_O	*Z* = 2
*M**_r_* = 607.06	*F*(000) = 634
Triclinic, *P*1	*D*_x_ = 1.278 Mg m^−^^3^
Hall symbol: -P 1	Mo *K*α radiation, λ = 0.71073 Å
*a* = 9.997 (2) Å	Cell parameters from 1416 reflections
*b* = 13.017 (3) Å	θ = 2.4–24.1°
*c* = 13.379 (3) Å	µ = 0.95 mm^−^^1^
α = 73.70 (3)°	*T* = 298 K
β = 77.95 (3)°	Block, colorless
γ = 72.25 (3)°	0.30 × 0.28 × 0.27 mm
*V* = 1577.0 (5) Å^3^	

### Data collection

**Table:**

Bruker SMART APEX CCD area-detector diffractometer	6300 independent reflections
Radiation source: fine-focus sealed tube	3199 reflections with *I* > 2σ(*I*)
graphite	*R*_int_ = 0.057
ω scans	θ_max_ = 26.5°, θ_min_ = 2.4°
Absorption correction: multi-scan (*SADABS*; Bruker, 2000)	*h* = −12→12
*T*_min_ = 0.764, *T*_max_ = 0.784	*k* = −16→16
12467 measured reflections	*l* = −16→16

### Refinement

**Table:**

Refinement on *F*^2^	Primary atom site location: structure-invariant direct methods
Least-squares matrix: full	Secondary atom site location: difference Fourier map
*R*[*F*^2^ > 2σ(*F*^2^)] = 0.081	Hydrogen site location: inferred from neighbouring sites
*wR*(*F*^2^) = 0.190	H-atom parameters constrained
*S* = 0.99	*w* = 1/[σ^2^(*F*_o_^2^) + (0.0763*P*)^2^] where *P* = (*F*_o_^2^ + 2*F*_c_^2^)/3
6300 reflections	(Δ/σ)_max_ < 0.001
346 parameters	Δρ_max_ = 0.51 e Å^−^^3^
18 restraints	Δρ_min_ = −0.37 e Å^−^^3^

### Special details

**Table:**

Geometry. All e.s.d.'s (except the e.s.d. in the dihedral angle between two l.s. planes) are estimated using the full covariance matrix. The cell e.s.d.'s are taken into account individually in the estimation of e.s.d.'s in distances, angles and torsion angles; correlations between e.s.d.'s in cell parameters are only used when they are defined by crystal symmetry. An approximate (isotropic) treatment of cell e.s.d.'s is used for estimating e.s.d.'s involving l.s. planes.
Refinement. Refinement of *F*^2^ against ALL reflections. The weighted *R*-factor *wR* and goodness of fit *S* are based on *F*^2^, conventional *R*-factors *R* are based on *F*, with *F* set to zero for negative *F*^2^. The threshold expression of *F*^2^ > σ(*F*^2^) is used only for calculating *R*-factors(gt) *etc*. and is not relevant to the choice of reflections for refinement. *R*-factors based on *F*^2^ are statistically about twice as large as those based on *F*, and *R*- factors based on ALL data will be even larger.

### Fractional atomic coordinates and isotropic or equivalent isotropic displacement parameters (Å^2^)

**Table:**

	*x*	*y*	*z*	*U*_iso_*/*U*_eq_	Occ. (<1)
Zn1	0.76677 (7)	0.52215 (5)	0.74443 (5)	0.0565 (3)	
S1	0.9762 (3)	0.7599 (2)	0.8261 (2)	0.1405 (10)	
S2	0.7074 (3)	0.9701 (2)	0.18236 (19)	0.1322 (9)	
O1	0.6935 (4)	0.3968 (3)	0.8352 (3)	0.0743 (12)	
O2	0.6337 (7)	0.2068 (5)	0.8807 (4)	0.113 (2)	
O3	0.7078 (5)	0.5764 (3)	0.6026 (3)	0.0751 (12)	
O4	0.5434 (6)	0.6530 (5)	0.4556 (3)	0.0952 (15)	
O5	0.9890 (14)	0.0589 (13)	0.7592 (16)	0.201 (8)	0.50
H5A	0.9333	0.0169	0.7815	0.241*	0.50
H5B	1.0545	0.0478	0.7084	0.241*	0.50
N1	0.5803 (6)	0.6341 (5)	0.8016 (4)	0.0714 (14)	
N2	0.5448 (7)	0.7845 (4)	0.5970 (4)	0.0893 (18)	
H2A	0.5726	0.7115	0.5985	0.107*	
H2B	0.5746	0.8205	0.5321	0.107*	
N3	0.9438 (6)	0.4057 (4)	0.6861 (5)	0.0735 (15)	
N4	0.9269 (6)	0.2291 (4)	0.8690 (4)	0.0778 (15)	
H4A	0.9646	0.1849	0.8236	0.093*	
H4B	0.8412	0.2707	0.8519	0.093*	
N5	0.8880 (6)	0.5913 (5)	0.7923 (5)	0.0799 (16)	
N6	0.6414 (11)	0.8946 (7)	0.3922 (8)	0.150 (3)	
C1	0.4525 (8)	0.4901 (9)	0.8824 (5)	0.085 (2)	
C2	0.5598 (8)	0.3956 (7)	0.8705 (4)	0.0709 (19)	
C3	0.5227 (10)	0.2965 (9)	0.8983 (5)	0.092 (2)	
C4	0.3836 (12)	0.2933 (12)	0.9369 (6)	0.125 (4)	
H4	0.3624	0.2250	0.9553	0.150*	
C5	0.2823 (13)	0.3802 (15)	0.9484 (8)	0.144 (6)	
H5	0.1903	0.3741	0.9736	0.172*	
C6	0.3115 (9)	0.4821 (11)	0.9230 (6)	0.116 (3)	
H6	0.2397	0.5451	0.9323	0.139*	
C7	0.6075 (13)	0.1001 (8)	0.9051 (9)	0.162 (5)	
H7A	0.5748	0.0793	0.9787	0.242*	
H7B	0.6936	0.0466	0.8876	0.242*	
H7C	0.5367	0.1025	0.8652	0.242*	
C8	0.4715 (8)	0.6024 (8)	0.8540 (5)	0.088 (2)	
H8	0.3958	0.6569	0.8765	0.105*	
C9	0.5723 (8)	0.7536 (6)	0.7858 (6)	0.091 (2)	
H9A	0.6326	0.7613	0.8299	0.109*	
H9B	0.4757	0.7932	0.8067	0.109*	
C10	0.6180 (9)	0.8039 (6)	0.6729 (6)	0.096 (2)	
H10A	0.6007	0.8832	0.6640	0.115*	
H10B	0.7193	0.7736	0.6565	0.115*	
C11	0.3859 (10)	0.8191 (7)	0.6135 (7)	0.114 (3)	
H11A	0.3522	0.7958	0.6867	0.171*	
H11B	0.3502	0.7854	0.5731	0.171*	
H11C	0.3535	0.8984	0.5914	0.171*	
C12	0.8471 (10)	0.4272 (7)	0.5243 (6)	0.085 (2)	
C13	0.7338 (8)	0.5214 (6)	0.5289 (5)	0.0712 (19)	
C14	0.6466 (9)	0.5591 (7)	0.4474 (5)	0.081 (2)	
C15	0.6657 (11)	0.5033 (9)	0.3720 (6)	0.104 (3)	
H15	0.6036	0.5282	0.3221	0.125*	
C16	0.7754 (14)	0.4109 (11)	0.3687 (8)	0.118 (4)	
H16	0.7890	0.3745	0.3152	0.142*	
C17	0.8643 (11)	0.3716 (7)	0.4418 (8)	0.105 (3)	
H17	0.9377	0.3078	0.4389	0.126*	
C18	0.4627 (10)	0.7033 (8)	0.3714 (6)	0.122 (3)	
H18A	0.5249	0.7180	0.3070	0.183*	
H18B	0.3979	0.7716	0.3832	0.183*	
H18C	0.4105	0.6543	0.3666	0.183*	
C19	0.9490 (8)	0.3819 (5)	0.5987 (7)	0.085 (2)	
H19	1.0291	0.3281	0.5803	0.103*	
C20	1.0633 (7)	0.3501 (6)	0.7456 (7)	0.097 (2)	
H20A	1.1159	0.4029	0.7418	0.117*	
H20B	1.1263	0.2913	0.7137	0.117*	
C21	1.0177 (7)	0.3020 (6)	0.8579 (7)	0.085 (2)	
H21A	1.1008	0.2603	0.8923	0.102*	
H21B	0.9667	0.3617	0.8926	0.102*	
C22	0.9086 (9)	0.1580 (7)	0.9783 (6)	0.105 (3)	
H22A	0.9993	0.1252	1.0028	0.158*	
H22B	0.8676	0.1005	0.9775	0.158*	
H22C	0.8473	0.2029	1.0243	0.158*	
C23	0.9247 (7)	0.6603 (6)	0.8069 (5)	0.0772 (18)	
C24	0.6730 (10)	0.9266 (7)	0.3022 (8)	0.112 (3)	

### Atomic displacement parameters (Å^2^)

**Table:**

	*U*^11^	*U*^22^	*U*^33^	*U*^12^	*U*^13^	*U*^23^
Zn1	0.0650 (5)	0.0525 (4)	0.0489 (4)	−0.0206 (3)	0.0020 (3)	−0.0083 (3)
S1	0.183 (2)	0.131 (2)	0.153 (2)	−0.0873 (19)	−0.0027 (18)	−0.0688 (17)
S2	0.178 (2)	0.1057 (17)	0.0884 (16)	−0.0077 (16)	−0.0165 (15)	−0.0174 (13)
O1	0.072 (3)	0.074 (3)	0.070 (3)	−0.034 (2)	−0.010 (2)	0.010 (2)
O2	0.139 (5)	0.099 (4)	0.120 (4)	−0.080 (4)	−0.067 (4)	0.036 (3)
O3	0.107 (3)	0.068 (3)	0.047 (2)	−0.025 (2)	0.001 (2)	−0.014 (2)
O4	0.133 (4)	0.111 (4)	0.050 (3)	−0.053 (4)	−0.013 (3)	−0.007 (3)
O5	0.117 (10)	0.167 (14)	0.36 (3)	−0.047 (10)	−0.001 (12)	−0.148 (16)
N1	0.074 (4)	0.090 (4)	0.044 (3)	−0.008 (3)	−0.009 (3)	−0.022 (3)
N2	0.131 (6)	0.059 (3)	0.068 (4)	−0.018 (4)	−0.021 (4)	−0.001 (3)
N3	0.073 (4)	0.063 (3)	0.081 (4)	−0.028 (3)	0.019 (3)	−0.021 (3)
N4	0.096 (4)	0.053 (3)	0.089 (4)	−0.024 (3)	−0.028 (3)	−0.007 (3)
N5	0.076 (4)	0.084 (4)	0.095 (4)	−0.036 (3)	0.004 (3)	−0.038 (3)
N6	0.199 (7)	0.121 (6)	0.124 (6)	−0.057 (5)	0.000 (5)	−0.018 (5)
C1	0.080 (6)	0.151 (8)	0.034 (3)	−0.047 (6)	−0.003 (3)	−0.021 (4)
C2	0.073 (5)	0.111 (6)	0.030 (3)	−0.046 (5)	−0.010 (3)	0.007 (3)
C3	0.114 (7)	0.130 (7)	0.047 (4)	−0.075 (6)	−0.033 (4)	0.021 (4)
C4	0.120 (8)	0.226 (13)	0.056 (5)	−0.121 (9)	−0.014 (6)	0.007 (6)
C5	0.105 (8)	0.31 (2)	0.069 (6)	−0.127 (11)	0.016 (6)	−0.059 (9)
C6	0.074 (5)	0.236 (12)	0.060 (5)	−0.057 (7)	0.012 (4)	−0.067 (6)
C7	0.233 (12)	0.125 (8)	0.174 (10)	−0.131 (8)	−0.104 (9)	0.040 (7)
C8	0.063 (4)	0.146 (8)	0.041 (4)	0.000 (5)	−0.004 (3)	−0.032 (4)
C9	0.113 (6)	0.079 (5)	0.076 (5)	0.007 (4)	−0.023 (4)	−0.039 (4)
C10	0.134 (7)	0.066 (4)	0.093 (6)	−0.021 (4)	−0.032 (5)	−0.021 (4)
C11	0.120 (7)	0.102 (6)	0.115 (7)	−0.005 (5)	−0.038 (5)	−0.029 (5)
C12	0.111 (6)	0.084 (5)	0.064 (4)	−0.053 (5)	0.032 (4)	−0.024 (4)
C13	0.095 (5)	0.070 (4)	0.052 (4)	−0.049 (4)	0.032 (4)	−0.019 (3)
C14	0.119 (6)	0.092 (5)	0.044 (4)	−0.062 (5)	0.012 (4)	−0.015 (4)
C15	0.143 (8)	0.137 (8)	0.058 (5)	−0.086 (7)	0.022 (5)	−0.037 (5)
C16	0.162 (10)	0.148 (9)	0.082 (6)	−0.103 (8)	0.051 (6)	−0.064 (7)
C17	0.134 (7)	0.093 (6)	0.088 (6)	−0.054 (5)	0.047 (6)	−0.043 (5)
C18	0.151 (7)	0.166 (9)	0.066 (5)	−0.080 (7)	−0.028 (5)	−0.003 (5)
C19	0.087 (5)	0.051 (4)	0.103 (6)	−0.031 (4)	0.042 (5)	−0.020 (4)
C20	0.056 (4)	0.068 (5)	0.156 (8)	−0.017 (4)	0.000 (5)	−0.018 (5)
C21	0.072 (4)	0.062 (4)	0.131 (7)	−0.022 (4)	−0.026 (4)	−0.022 (4)
C22	0.139 (7)	0.089 (5)	0.085 (5)	−0.028 (5)	−0.047 (5)	0.004 (4)
C23	0.077 (5)	0.085 (5)	0.072 (4)	−0.022 (4)	−0.002 (3)	−0.029 (4)
C24	0.166 (8)	0.074 (5)	0.092 (6)	−0.041 (5)	−0.002 (6)	−0.015 (5)

### Geometric parameters (Å, °)

**Table:**

Zn1—O3	1.977 (4)	C5—H5	0.9300
Zn1—O1	1.979 (4)	C6—H6	0.9300
Zn1—N5	2.001 (6)	C7—H7A	0.9600
Zn1—N3	2.119 (5)	C7—H7B	0.9600
Zn1—N1	2.135 (5)	C7—H7C	0.9600
S1—C23	1.632 (8)	C8—H8	0.9300
S2—C24	1.545 (10)	C9—C10	1.503 (10)
O1—C2	1.325 (7)	C9—H9A	0.9700
O2—C3	1.381 (10)	C9—H9B	0.9700
O2—C7	1.426 (9)	C10—H10A	0.9700
O3—C13	1.314 (7)	C10—H10B	0.9700
O4—C14	1.353 (9)	C11—H11A	0.9600
O4—C18	1.412 (9)	C11—H11B	0.9600
O5—H5A	0.8500	C11—H11C	0.9600
O5—H5B	0.8500	C12—C13	1.399 (10)
N1—C8	1.280 (9)	C12—C17	1.437 (11)
N1—C9	1.489 (9)	C12—C19	1.450 (11)
N2—C10	1.483 (9)	C13—C14	1.423 (10)
N2—C11	1.499 (10)	C14—C15	1.351 (10)
N2—H2A	0.9000	C15—C16	1.363 (13)
N2—H2B	0.9000	C15—H15	0.9300
N3—C19	1.278 (9)	C16—C17	1.347 (13)
N3—C20	1.460 (9)	C16—H16	0.9300
N4—C21	1.462 (8)	C17—H17	0.9300
N4—C22	1.503 (9)	C18—H18A	0.9600
N4—H4A	0.9000	C18—H18B	0.9600
N4—H4B	0.9000	C18—H18C	0.9600
N5—C23	1.144 (8)	C19—H19	0.9300
N6—C24	1.168 (10)	C20—C21	1.489 (10)
C1—C2	1.387 (10)	C20—H20A	0.9700
C1—C6	1.425 (11)	C20—H20B	0.9700
C1—C8	1.465 (11)	C21—H21A	0.9700
C2—C3	1.380 (10)	C21—H21B	0.9700
C3—C4	1.388 (12)	C22—H22A	0.9600
C4—C5	1.290 (16)	C22—H22B	0.9600
C4—H4	0.9300	C22—H22C	0.9600
C5—C6	1.380 (15)		
			
O3—Zn1—O1	114.77 (18)	C10—C9—H9A	109.4
O3—Zn1—N5	122.2 (2)	N1—C9—H9B	109.4
O1—Zn1—N5	123.0 (2)	C10—C9—H9B	109.4
O3—Zn1—N3	89.1 (2)	H9A—C9—H9B	108.0
O1—Zn1—N3	88.67 (19)	N2—C10—C9	114.0 (7)
N5—Zn1—N3	93.1 (2)	N2—C10—H10A	108.8
O3—Zn1—N1	89.57 (18)	C9—C10—H10A	108.8
O1—Zn1—N1	89.0 (2)	N2—C10—H10B	108.8
N5—Zn1—N1	90.4 (2)	C9—C10—H10B	108.8
N3—Zn1—N1	176.5 (2)	H10A—C10—H10B	107.7
C2—O1—Zn1	128.0 (4)	N2—C11—H11A	109.5
C3—O2—C7	118.9 (8)	N2—C11—H11B	109.5
C13—O3—Zn1	128.1 (4)	H11A—C11—H11B	109.5
C14—O4—C18	116.7 (7)	N2—C11—H11C	109.5
H5A—O5—H5B	120.0	H11A—C11—H11C	109.5
C8—N1—C9	116.2 (6)	H11B—C11—H11C	109.5
C8—N1—Zn1	122.4 (5)	C13—C12—C17	118.7 (9)
C9—N1—Zn1	121.3 (5)	C13—C12—C19	123.6 (7)
C10—N2—C11	116.9 (6)	C17—C12—C19	117.7 (9)
C10—N2—H2A	108.1	O3—C13—C12	123.0 (7)
C11—N2—H2A	108.1	O3—C13—C14	119.9 (7)
C10—N2—H2B	108.1	C12—C13—C14	117.1 (7)
C11—N2—H2B	108.1	C15—C14—O4	124.8 (8)
H2A—N2—H2B	107.3	C15—C14—C13	122.1 (9)
C19—N3—C20	118.3 (7)	O4—C14—C13	113.0 (6)
C19—N3—Zn1	121.3 (5)	C14—C15—C16	120.4 (10)
C20—N3—Zn1	120.4 (5)	C14—C15—H15	119.8
C21—N4—C22	113.0 (6)	C16—C15—H15	119.8
C21—N4—H4A	109.0	C17—C16—C15	120.8 (9)
C22—N4—H4A	109.0	C17—C16—H16	119.6
C21—N4—H4B	109.0	C15—C16—H16	119.6
C22—N4—H4B	109.0	C16—C17—C12	120.8 (9)
H4A—N4—H4B	107.8	C16—C17—H17	119.6
C23—N5—Zn1	157.9 (6)	C12—C17—H17	119.6
C2—C1—C6	120.1 (9)	O4—C18—H18A	109.5
C2—C1—C8	124.6 (6)	O4—C18—H18B	109.5
C6—C1—C8	115.3 (9)	H18A—C18—H18B	109.5
O1—C2—C3	119.7 (8)	O4—C18—H18C	109.5
O1—C2—C1	123.2 (7)	H18A—C18—H18C	109.5
C3—C2—C1	117.1 (8)	H18B—C18—H18C	109.5
C2—C3—O2	113.9 (7)	N3—C19—C12	128.9 (7)
C2—C3—C4	120.6 (11)	N3—C19—H19	115.5
O2—C3—C4	125.5 (9)	C12—C19—H19	115.5
C5—C4—C3	123.3 (12)	N3—C20—C21	112.5 (6)
C5—C4—H4	118.4	N3—C20—H20A	109.1
C3—C4—H4	118.4	C21—C20—H20A	109.1
C4—C5—C6	119.4 (11)	N3—C20—H20B	109.1
C4—C5—H5	120.3	C21—C20—H20B	109.1
C6—C5—H5	120.3	H20A—C20—H20B	107.8
C5—C6—C1	119.5 (11)	N4—C21—C20	111.9 (6)
C5—C6—H6	120.2	N4—C21—H21A	109.2
C1—C6—H6	120.2	C20—C21—H21A	109.2
O2—C7—H7A	109.5	N4—C21—H21B	109.2
O2—C7—H7B	109.5	C20—C21—H21B	109.2
H7A—C7—H7B	109.5	H21A—C21—H21B	107.9
O2—C7—H7C	109.5	N4—C22—H22A	109.5
H7A—C7—H7C	109.5	N4—C22—H22B	109.5
H7B—C7—H7C	109.5	H22A—C22—H22B	109.5
N1—C8—C1	127.5 (7)	N4—C22—H22C	109.5
N1—C8—H8	116.3	H22A—C22—H22C	109.5
C1—C8—H8	116.3	H22B—C22—H22C	109.5
N1—C9—C10	111.2 (5)	N5—C23—S1	179.2 (7)
N1—C9—H9A	109.4	N6—C24—S2	177.3 (10)

### Hydrogen-bond geometry (Å, °)

**Table:**

*D*—H···*A*	*D*—H	H···*A*	*D*···*A*	*D*—H···*A*
N4—H4B···O2	0.90	2.38	3.001 (8)	126
N4—H4B···O1	0.90	1.84	2.682 (7)	155
N4—H4A···O5	0.90	1.99	2.839 (14)	157
N2—H2B···N6	0.90	1.93	2.834 (11)	179
N2—H2A···O4	0.90	2.35	2.889 (8)	119
N2—H2A···O3	0.90	1.86	2.691 (7)	153
